# Retraction: MicroRNA-27 Inhibits Autophagy and Promotes Proliferation of Multiple Myeloma Cells by Targeting the NEDD4/Notch1 Axis

**DOI:** 10.3389/fonc.2021.840086

**Published:** 2021-12-30

**Authors:** Frontiers Editorial Office

The journal and Chief Editors retract the 11 November 2020 article cited above.

Following publication, concerns were raised regarding the validity of the data in the article. The authors failed to provide the raw data or a satisfactory explanation during the investigation, which was conducted in accordance with Frontiers’ policies. Given the concerns, and the lack of raw data, the editors no longer have confidence in the findings presented in the article.

This retraction was approved by the Chief Editors of Frontiers in Oncology and the Chief Executive Editor of Frontiers. The authors have not responded to any correspondence regarding the retraction.

